# Apicoplast Dynamics During *Plasmodium* Cell Cycle

**DOI:** 10.3389/fcimb.2022.864819

**Published:** 2022-04-29

**Authors:** Arwa Elaagip, Sabrina Absalon, Anat Florentin

**Affiliations:** ^1^ Department of Parasitology and Medical Entomology, Faculty of Medical Laboratory Sciences, University of Khartoum, Khartoum, Sudan; ^2^ Department of Pharmacology and Toxicology, Indiana University School of Medicine, Indianapolis, IN, United States; ^3^ The Kuvin Center for the Study of Infectious and Tropical Diseases, Department of Microbiology and Molecular Genetics, Institute for Medical Research Israel-Canada, Faculty of Medicine, Hebrew University of Jerusalem, Jerusalem, Israel

**Keywords:** plasmodium, malaria, apicoplast, cell cycle, schizogony, organelle dynamics

## Abstract

The deadly malaria parasite, *Plasmodium falciparum*, contains a unique subcellular organelle termed the apicoplast, which is a clinically-proven antimalarial drug target. The apicoplast is a plastid with essential metabolic functions that evolved *via* secondary endosymbiosis. As an ancient endosymbiont, the apicoplast retained its own genome and it must be inherited by daughter cells during cell division. During the asexual replication of *P. falciparum* inside human red blood cells, both the parasite, and the apicoplast inside it, undergo massive morphological changes, including DNA replication and division. The apicoplast is an integral part of the cell and thus its development is tightly synchronized with the cell cycle. At the same time, certain aspects of its dynamics are independent of nuclear division, representing a degree of autonomy in organelle biogenesis. Here, we review the different aspects of organelle dynamics during *P. falciparum* intraerythrocytic replication, summarize our current understanding of these processes, and describe the many open questions in this area of parasite basic cell biology.

## Introduction

### The Study of Fundamental Cell Biology of Malaria Parasites

Malaria is a worldwide leading cause of morbidity and mortality, infecting predominantly people in tropical and sub-tropical regions. In 2020, WHO estimated 250 million malaria cases and reported about 627000 deaths preponderantly in sub-Saharan African countries (World Health Organization, 2021). Malaria is caused by eukaryotic parasites of the genus *Plasmodium*, which are transmitted by female *Anopheles* mosquitoes (Garrido-Cardenas et al., 2019). It is primarily one species, *P. falciparum*, that is responsible for most of the mortality (World Health Organization, 2021). As yet, there are no effective vaccines and the parasite gained resistance to all clinically available antimalarial drugs, jeopardizing the progress that has been made in the last decade (Dondorp et al., 2009; Phillips et al., 2017; World Health Organization, 2019). Since the genome sequencing of *P. falciparum* in 2002 (Gardner et al., 2002), various surveys on population genetics, genomics, transcriptomics and proteomics brought into view the potential of targeting parasite-specific molecular pathways in eliminating malaria (Winzeler, 2008; Su et al., 2019). These strategies rely heavily on advanced techniques in cell biology and molecular genetics including various conditional-knockdown methods and the application of CRISPR/Cas9 genome engineering (Ghorbal et al., 2014; Wagner et al., 2014) [extensively reviewed in (Kudyba et al., 2021)]. Furthermore, a recently developed microscopy technique named ultrastructure expansion microscopy, allows the visualization of preserved expanded organelles with a 4-fold isotropic size increase, giving the resolution needed to monitor organelle dynamics of the microscopic malaria parasite during its replication (Bertiaux et al., 2021; Liffner and Absalon, 2021; Tomasina et al., 2021). Additional advanced microscopy techniques such as lattice light-sheet microscopy (LLSM) provide high-resolution in time and space that was used to determine the kinetics of parasite invasion into the erythrocyte and revealed detailed events in membrane remodeling (Geoghegan et al., 2021). Finally, some of the most advanced techniques in electron microscopy, for example Focused Ion Beam-Scanning Electron Microscopy (FIB-SEM), are used to gain the ultrastructure of nuclear division and subcellular organelle organization (Medeiros et al., 2012; Rudlaff et al., 2020). In this mini review, we describe *P. falciparum* mode of division in erythrocytes with a focus on a specialized plastid organelle named the apicoplast. This parasite-specific organelle, which is clinically targeted by various antimalarials, is essential for parasite viability due to its metabolic functions. Here we describe various open questions related to apicoplast biogenesis and discuss future research directions to examine the autonomous nature of apicoplast growth, fission, and segregation during *Plasmodium* cell division.

### *Plasmodium’s* Cell Cycle and Division

**Figure 1:**
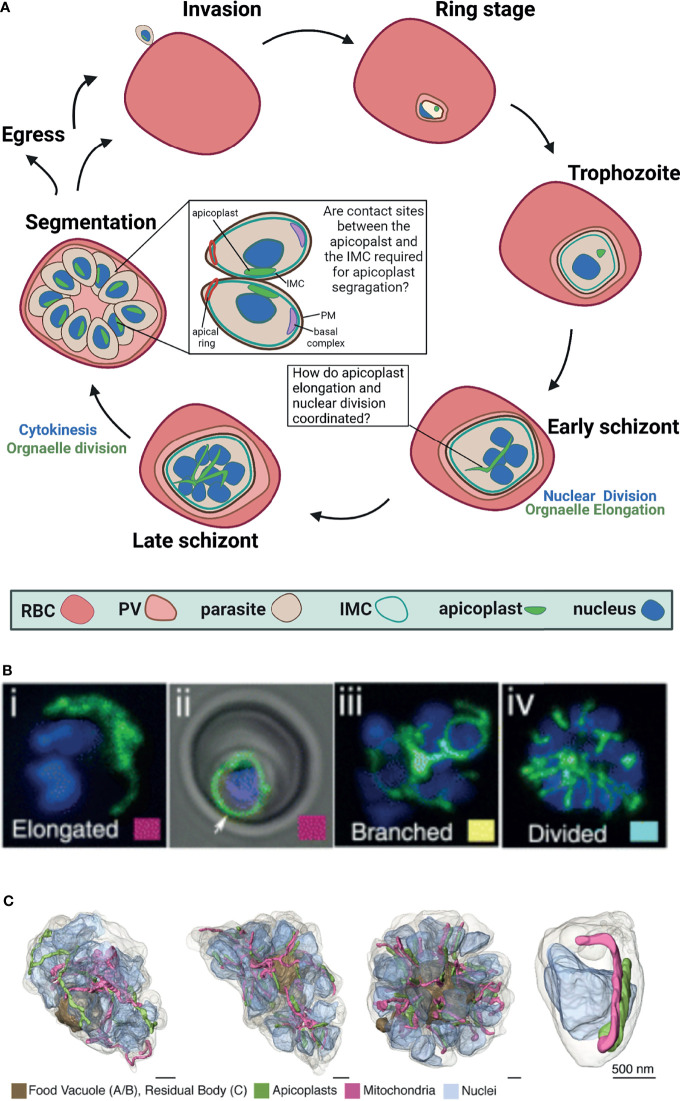
Apicoplast dynamics during intraerythrocytic development of *Plasmodium falciparum.* **(A)** Schematic representation of apicoplast dynamics during intraerythrocytic asexual replication of *P. falciparum*. RBC, red blood cell; PV, parasitophorous vacuole; IMC, inner membrane complex, PM, plasma membrane Created with *BioRender.com*. **(B)** Fluorescent microcopy of live apicoplast-tagged parasites (ACP-DsRed) co-labelled with a nuclear Hoechst dye. Apicoplast morphology was categorized into four groups based on developmental stage: (i) rounded (ii) elongated (iii) branched and (iv) divided. Image taken from van Dooren et al. (2005). **(C)**. Apicoplast morphologies throughout segmentation. By mid-segmentation, apicoplasts have divided to form one organelle for each nascent daughter cell. Ultra-resolution images were obtained using FIB-SEM, taken from Rudlaff et al. (2020).

The asexual replication of *P. falciparum* inside the red blood cell (RBC) begins with invasion by a single parasite into the host RBC and culminates 48 hours later in the egress of roughly 30 new daughter parasites (Garg et al., 2015). During this life cycle, the parasite grows and at a certain point begins a unique form of cell division called schizogony (Striepen et al., 2007; Francia and Striepen, 2014; Gubbels et al., 2021) (**Figure 1**). In this process, the parasite replicates its DNA and then follows with nuclear division inside an intact nuclear envelope to produce two nuclei (Absalon, 2020). This process is repeated multiple times asynchronously and produces a multi-nucleated cell (Gerald et al., 2011). Schizogony concludes in a single cytokinesis event, during which the multi-nucleated cell segments into ~30 daughter cells called merozoites that will egress and invade new host RBCs. This particular type of segmentation involves a membranous structure called the inner membrane complex (IMC) (Dearnley et al., 2012; Harding and Meissner, 2014). The IMC is a double lipid bilayer formed from a patchwork of flattened membrane vesicles that, together with associated proteins, lies closely underneath the parasite plasma membrane (Morrissette and Sibley, 2002; Kono et al., 2013). Beneath the IMC lies a network of alveolins—intermediate filament-like proteins that provide support to the IMC, and are common to all protists in the infrakingdom *Alveolata*, to which *Plasmodium* and other apicomplexan parasites belong (Khater et al., 2004; Gould et al., 2008; Al-Khattaf et al., 2014; Tremp et al., 2014). The IMC is involved in many essential parasite-specific functions including host cell invasion by anchoring many of the glideosome proteins required for actinomyosin-based gliding motility (Bergman et al., 2003; Keeley and Soldati, 2004; Baum et al., 2006; Frénal et al., 2010). Studies in the related parasite *Toxoplasma gondii* showed that the IMC defines the shape and structural stability of the parasite and is essential for parasite cell division (Mann and Beckers, 2001; Hu et al., 2002; Beck et al., 2010). In *P. falciparum*, it was shown that the IMC is associated with proteins inside the parasite and dictates the shape and rigidity of nascent merozoites, however, its biogenesis and mechanisms of action remain poorly understood (Absalon et al., 2016). Notably, it remains elusive how asynchronous nuclear replication coincides with a single event of segmentation where sub-cellular content must be equally distributed within daughter cells. The partitioning and distribution of organelles such as the endoplasmic reticulum, the Golgi apparatus and parasite-specific secretory organelles, are coordinated with segmentation (van Dooren et al., 2005). The fact that all these membranous compartments can be synthesized *de novo*, suggests some flexibility in their distribution to daughter cells which, in theory, can replenish the secretory pathway using inherited material as well as *de novo* synthesis. The case is different however for the parasite’s two endosymbiotic organelles, the mitochondrion and the apicoplast, which carry their own ancestral genomes. Unlike many other eukaryotic cells, the parasite carries only a single copy of each organelle that must be inherited accurately during cell division. As will be discussed below, the apicoplast organelle undergoes drastic morphological changes culminating in its division and sorting, while being coordinated with the different phases of schizogony.

### The Apicoplast, an Ancient Endosymbiont

*Plasmodium falciparum* is a remarkably complex unicellular parasite that, in addition to a mitochondrion, contains a second endosymbiont known as the apicoplast (McFadden et al., 1996; Köhler et al., 1997). This unique organelle evolved *via* a two-step endosymbiosis (Köhler et al., 1997). In the primary endosymbiotic event, a cyanobacterium was incorporated into a eukaryotic cell to form the modern chloroplast. During the second endosymbiotic event, a photosynthetic red alga was taken up by a protist, which led to the formation of a secondary plastid (van Dooren and Striepen, 2013). The subsequent evolution of the apicoplast resulted in the loss of all photosynthetic abilities, but retained important prokaryotic metabolic pathways including the synthesis of isoprenoids, fatty acids, iron-sulfur clusters, and heme (Ralph et al., 2004; Yeh and DeRisi, 2011; van Dooren and Striepen, 2013; Swift et al., 2021). In sharp contrast to its human host, the *Plasmodium* apicoplast shares molecular features with prokaryotes, plants and parasites, and therefore encompasses multiple parasite-specific drug targets (Fichera and Roos, 1997; Dahl and Rosenthal, 2007; Amberg-Johnson et al., 2017; Florentin et al., 2020). Despite its central cellular functions and clinical significance, little is known about the molecular mechanisms governing apicoplast biogenesis and development throughout the complex parasite’s cell cycle. The *Plasmodium* cell contains a single apicoplast organelle in all the different stages, and fusion and fission events that are common for other endosymbionts were never observed. However, the apicoplast undergoes striking morphological changes during the parasite’s intraerythrocytic growth, and develops from a small globular organelle into an elongated and branched structure (**Figure 1**). Moreover, the apicoplast cannot be synthesized *de novo*, and must be inherited. Therefore, the precise division of a single apicoplast into multiple organelles and their accurate segregation into the merozoites daughter cells are required to ensure that each nascent parasite contains a single complete apicoplast (**Figure 1**).

## Apicoplast Elongation During Nuclear Division

Right after invasion into the RBC in the early ring stages, the apicoplast is found as a single, small globular shape (McFadden et al., 1996; Lim and McFadden, 2010). It remains this way during most of the parasite’s intraerythrocytic development including the trophozoite stages, up until schizogony begins. Only after the onset of the asynchronous nuclear divisions, the apicoplast begins to elongate (van Dooren et al., 2005). During the first rounds of nuclear replication and division (2-5 nuclei), the apicoplast organelle begins to elongate at a fast speed. It is estimated that the replication of the organelle genome begins more or less during this phase of early schizogony, but clear evidence is still missing (Williamson et al., 2002). It is clear, however, that the replication of the apicoplast genome is key to organelle inheritance and involves prokaryotic machinery (as demonstrated by its antibiotic-sensitivity (Milton and Nelson, 2016)) as well as eukaryotic components such as autophagy related protein 8 (ATG8) (Walczak et al., 2018). As was demonstrated by fluorescence *in situ* hybridization, ATG8 mutants fail to pass on apicoplast genome to their daughter cells, representing a unique, parasite-specific adaptation to conserved eukaryotic factors (Walczak et al., 2018). As seen in **Figure 1**, while schizogony proceeds (5-10 nuclei) the organelle branches out to form an intricate structure, spanning throughout the cell volume (van Dooren et al., 2005). The molecular mechanisms underlying these drastic morphological changes are completely unknown, and to date, not a single gene was shown to be directly involved in this process. Interestingly, cdc2-related protein kinase 4 (CRK4), the cell-cycle regulator that controls the decision to undergo the first round of nuclear DNA replication, does not regulate organelle development (Ganter et al., 2017). In CRK4 mutants the apicoplast elongates and branches indistinctively from wild type parasites, despite the fact that nuclear DNA replication is completely blocked (Ganter et al., 2017). This intriguing observation suggests that although apicoplast development is synced with schizogony, it is not dependent on nuclear replication. Alternatively, it may be that apicoplast and nuclear division are coupled in wildtype parasites but decoupled upon CRK4 knockdown. Additional experimentation is required to distinguish between these possibilities, and to test whether apicoplast elongation and branching is regulated by an autonomous, organelle-specific mechanism that does not rely on known cell cycle checkpoints. What is that mechanism, and how is overall cellular synchrony maintained remains to be investigated.

## Apicoplast Division

The apicoplast elongates and branches, and reaches its most intricate structure during early segmentation (Rudlaff et al., 2020). As described above, segmentation is the *Plasmodium* equivalence of cytokinesis, and in this process the multinucleated schizont is separated into multiple daughter cells called merozoites. It is interesting to note that while the merozoites have already started individualization, the apicoplast still exists as a single organelle. Recent ultra-resolution studies revealed that it is only during mid segmentation that the apicoplast divides to produce daughter organelles (Rudlaff et al., 2020). This organelle division (or fission) needs to accurately result in a single apicoplast for each individual nascent merozoite (**Figure 1**). Because nuclear divisions are asynchronous and schizogony can produce varying numbers of daughter merozoites, it is unclear how apicoplast fission is regulated to produce the right number of organelles. It is clear though that at the end of this process each new daughter merozoite is equipped with a single new apicoplast organelle. The division machinery itself is unknown. The endosymbiotic evolution of the apicoplast suggests that this machinery may involve prokaryotic as well as eukaryotic components that have mostly remained elusive. The binary bacterial division involves an ancestral machinery that is based on a small GTPase called FtsZ that forms a contractile structure called a Z-ring at the fission site (Barrows and Goley, 2021). Plant chloroplasts also use an FtsZ homolog that forms a Z-ring at their inner membrane, which then recruits additional contracting proteins to the organelle outer membrane (TerBush et al., 2013). The outer ring is formed by eukaryotic dynamin-like proteins, and thus, the binary chloroplast division machinery progresses through reciprocal communication between inside and outside protein complexes across the two organellar membranes (Miyagishima, 2017). Despite the common origin, apicoplast division differs from those of its bacterial and chloroplast ancestors in several ways. First of all, it is not a binary division (i.e. a single organelle/cell splitting into two), rather a partitioning of a very long structure into multiple organelles, probably involving numerous fission sites. Second, unlike bacteria and chloroplasts, fission does not occur across one or two membranes, rather involves the contraction of four lipid bilayers. And most importantly, the *Plasmodium* genome does not encode any homologs to components of the FtsZ division machinery, suggesting that the apicoplast divergently evolved a distinct mode of division (Vaishnava et al., 2005; Dooren et al., 2006; Verhoef et al., 2021).

As stated above, in plants dynamin-like proteins contract the outer membrane and interact with the FtsZ ring in the inner membranes to facilitate chloroplast division (Miyagishima, 2017). Dynamins are large GTPases that mediate membrane remodeling and, similar to FtsZ, form ring-like structures in eukaryotic systems (Jimah and Hinshaw, 2019). In mammalian and yeast cells, dynamin-related proteins mediate mitochondrial fission (Nottia et al., 2021). Both *Plasmodium* and the related parasite *Toxoplasma* encode three dynamin-related genes that seem to diverge significantly from the chloroplasts orthologs and are more similar to the mitochondrial dynamin (Li et al., 2004; Charneau et al., 2007; Breinich et al., 2009; Heredero-Bermejo et al., 2019). A study in *T. gondii* revealed a role for a dynamin-related protein in apicoplast division (van Dooren et al., 2009). It remains to be investigated whether the *Plasmodium* orthologue is similarly involved in apicoplast division, whether a dynamin-ring is formed and whether other contractile rings are formed during this elusive process.

## Apicoplast Segregation Into Daughter Merozoites

The segmentation of the multinucleated cell into dozens of merozoite daughter cells involves several parasite-specific cellular structures that control and facilitate this complicated process. The inner membrane complex (IMC), the associated basal complex, and the interaction of these structures with parasite nuclei are critical for segmentation. As described above, the IMC is a unique membranous structure with associated proteins inside the parasite that dictates its shape and rigidity (Beck et al., 2010; Dearnley et al., 2012). At the onset of segmentation, a ring of IMC proteins moves from the apical to the basal end of the nascent merozoite, leaving behind the incorporated IMC proteins that form a cylinder-like structure around its contents (Tran et al., 2010). At the apical end of the merozoite, the apical ring is hypothesized to nucleate the formation of sub-pellicular microtubules and that this polymerization may facilitate IMC progression from the apical to basal end (Pacheco et al., 2020). The basal complex is a group of proteins at the posterior end of the IMC, hypothesized to generate force to pull the IMC down the length of the daughter cell and mediate the final abscission step of cytokinesis (Rudlaff et al., 2019). Together, the IMC and basal complex orchestrate daughter parasite assembly and division through critical interactions with the parasite nuclei (Engelberg et al., 2016; Morano and Dvorin, 2021). Not only that IMC biogenesis and its mechanisms of action remain poorly understood; it is also completely unclear whether and how it is involved in accurate organelle sorting into daughter cells. Ultrastructure studies demonstrate that the apicoplast divides only after segmentation begins, when the IMC is roughly halfway through the cell volume (Rudlaff et al., 2020). At the end of the IMC movement, after daughter cells have been individualized, each one of them will also have a single apicoplast. How are the multiple daughter organelles sorted accurately between the nascent cells? It is particularly intriguing because nuclear divisions are asynchronous and can result in a different number of daughter cells. In *Toxoplasma gondii*, apicoplast division was shown to be associated with the centrosome (Striepen et al., 2000). Although *Toxoplasma* centrosome differs significantly from the *Plasmodium* microtubule organizing center in architecture and organization, they might serve a similar function with respect to apicoplast segregation. It is tempting to speculate that accurate organelle segregation during late schizogony is mediated through interactions between the apicoplast and the IMC. The rationale behind this hypothesis is that in the last decade, functional tethering between organelles has been described in most cellular eukaryotic systems, underlying the physiological significance of such interactions (Scorrano et al., 2019). Moreover, early ultrastructural analysis suggested contact sites between the *Plasmodium* ER and the apicoplast (Hopkins et al., 1999), as well as more recent observations between these organelles in *T. gondii* (Tomova et al., 2009). Similarly, contact sites between the mitochondrion and the apicoplast were reported (van Dooren et al., 2005), and it was suggested that these interactions might represent a mechanism to ensure accurate sorting of organelles (Verhoef et al., 2021). Therefore, it may very well be that the IMC serves as a central cellular hub that physically links the nuclei and organelles including the apicoplast during segmentation, to ensure that every daughter parasite receives a complete set of cellular content. This hypothesis needs to be tested experimentally, and if proven correct, will explain how accurate organelle sorting is achieved despite the asynchronous nature of nuclear division. Such physical tethering will also provide unique evidence of functional organelle contact sites in *Plasmodium*.

## Apicoplast Dynamics in Other *Plasmodium* Life Stages

Due to limited culturing and complicated experimental settings, the study of apicoplast dynamics in *P. falciparum* has been mostly focused on the parasite’s asexual replication within the erythrocyte. A small subpopulation of the erythrocyte infecting parasites will undergo sexual differentiation in a process called gametocytogenesis, transforming into the infective gametocyte stages (Ngotho et al., 2019). The metabolic function of the apicoplast during this process is comparable to its roles during the intraerythrocytic asexual replication (Wiley et al., 2015), and the morphological changes that it undergoes were described by live microscopy (Okamoto et al., 2009). A detailed description and the molecular mechanisms underlying the timing and morphological transitions of apicoplast biogenesis and fission events are yet to be unveiled.

Most importantly, the complete parasite life cycle also includes massive replication in human hepatocytes as well as sexual development within the mosquito vector. The studies of organelle dynamics during these stages rely mostly on murine models, involving related *Plasmodium* species such as *P. berghei* (Stanway et al., 2009). The imaging of *P. berghei*-infecting mouse hepatocytes revealed remarkably complex organelle morphologies (Stanway et al., 2011). In these stages, a single infecting sporozoite divides and develops into thousands of daughter merozoites inside the hepatocyte, and the rapid growth and fission of the apicoplast during this process is astonishing (Stanway et al., 2011). These processes are reminiscent of the morphological changes that the apicoplast undergoes inside the erythrocyte but on a much larger scale, and are even less understood. Similarly, very little data have been gathered concerning apicoplast development during mosquito stages, which are characterized by additional metabolic requirements from the organelle (van Schaijk et al., 2013; Korbmacher et al., 2021). The questions remain whether similar processes occur during the apicoplast liver and mosquito development in *P. falciparum*, and what are the molecular mechanisms that control and execute these subcellular developments.

## Conclusions and Open Questions

- **The autonomous nature of apicoplast development during nuclear replication:** Although apicoplast elongation happens together with nuclear replication, these two processes seem to occur independently of each other, as suggested by the normal apicoplast development documented in cell cycle mutants (Ganter et al., 2017), and the normal asexual replication observed in apicoplast-less parasites supplemented with essential metabolites (Yeh and DeRisi, 2011; Florentin et al., 2020). How is organelle biogenesis coordinated with nuclear replication and division? Is there an apicoplast-specific mechanism in the organelle itself that times and controls these morphological changes? If so, what is it? What other regulatory mechanisms are in place to ensure that nuclear replication and organelle development are coordinated?
- **Obscure aspects of apicoplast fission:** Despite the common origin, apicoplast division differs from those of bacteria and chloroplasts because it is not a binary division, it occurs across four lipid bilayers, and it does not involve a homolog of the FtsZ division machinery (Vaishnava et al., 2005; Dooren et al., 2006; Verhoef et al., 2021). All of these facts suggest that the apicoplast divergently evolved a distinct mode of division, which might represent an attractive target for drug development. What cellular components mediate this process? Are dynamin-related-proteins involved in *Plasmodium* apicoplast fission, similar to those that mediate this process in *Toxoplasma*? If so, on which of the four membranes do they act, and what other organellar components contract the membranes from within?
- **Apicoplast-cytoskeleton contact sites during segmentation:** After the apicoplast divides, the resulting multiple organelles are sorted accurately between the daughter merozoite cells. It is unclear how this exact sorting is mediated, particularly in light of the asynchronous nature of nuclear divisions that result in varying numbers of daughter cells. One intriguing hypothesis is that physical contact sites between the apicoplast and other cellular components guarantee precise division. The inner membrane complex (IMC) is a key mediator of segmentation, and thus might serve as a central tethering point for cellular content, including the apicoplast. Experimental evidence is still missing, and thus the process of accurate organelle sorting at the final stage of the *Plasmodium* cell cycle remains enigmatic.

## Author Contributions

AE, SA, and AF wrote together the manuscript. All authors contributed to manuscript revision, read, and approved the submitted version.

## Acknowledgments

AF is supported by The Abisch-Frenkel Faculty Development Lectureship. Manuscript preparation was supported by a training-travel grant by The Kuvin Foundation to AE.

## Conflict of Interest

The authors declare that the research was conducted in the absence of any commercial or financial relationships that could be construed as a potential conflict of interest.

## Publisher’s Note

All claims expressed in this article are solely those of the authors and do not necessarily represent those of their affiliated organizations, or those of the publisher, the editors and the reviewers. Any product that may be evaluated in this article, or claim that may be made by its manufacturer, is not guaranteed or endorsed by the publisher.
